# The Dual Effects of CDK4/6 Inhibitors on Tumor Immunity

**DOI:** 10.3390/cancers17243997

**Published:** 2025-12-15

**Authors:** Yiran Si, Hongli Li, Yehui Shi

**Affiliations:** 1 Department of Phase I Clinical Trial, Tianjin Medical University Cancer Institute & Hospital, National Clinical Research Center for Cancer, Tianjin’s Clinical Research Center for Cancer, Key Laboratory of Breast Cancer Prevention and Therapy, Key Laboratory of Cancer Immunology and Biotherapy, Tianjin 300060, China; siyiran@tjmuch.com (Y.S.); lihongli@tjmuch.com (H.L.); 2 Department of Breast Oncology, Tianjin Medical University Cancer Institute & Hospital, National Clinical Research Center for Cancer, Tianjin’s Clinical Research Center for Cancer, Key Laboratory of Breast Cancer Prevention and Therapy, Key Laboratory of Cancer Immunology and Biotherapy, Tianjin 300060, China

**Keywords:** CDK4/6 inhibitors, tumor immunity, immune microenvironment, dual effects, immunotherapy

## Abstract

CDK4/6 inhibitors exert effective anti-tumor effects by blocking the cell cycle and, as a result, have become vital in the systemic treatment of malignant tumors. Previous research has indicated that CDK4/6 inhibitors not only exert effects on the cell cycle but also have regulatory roles in tumor immunity, although the research findings are controversial. This study comprehensively summarizes the molecular mechanisms by which CDK4/6 inhibitors activate or suppress anti-tumor immunity and reveals the dual effects of CDK4/6 inhibitors on influencing interferon signaling, mediating senescence, and altering certain immune cells. In addition, the results of clinical trials of CDK4/6 inhibitors combined with immunotherapy are thought-provoking, with the severe adverse events that occur after treatment being the main factor affecting their therapeutic effect. Therefore, the future direction of this combined treatment strategy deserves further exploration.

## Simple Summary

An increasing number of studies have shown that CDK4/6 inhibitors not only act on the cell cycle but also have an impact on tumor immunity. This review summarized the preclinical and clinical trials results of CDK4/6 inhibitors on tumor immunity. It proposed for the first time that CDK4/6 inhibitors exhibit a “duality” in activating or inhibiting anti-tumor immunity when interfering with the interferon signaling pathway, inducing cellular senescence, and certain specific immune cell subsets. It also provided sufficient theoretical bases and future development directions for the combination of CDK4/6 inhibitors and immunotherapy.

## 1. Introduction

Cell cycle-dependent kinase 4/6 (CDK4/6) is a central protein in modulating the cell cycle. CDK4/6 binds with cyclin D and phosphorylates retinoblastoma (Rb) protein, which promotes the transition of the cell cycle and stimulates cell proliferation [1]. In the occurrence and development of malignant tumors, overactivation of upstream signaling pathways or defects in the genes encoding cyclin D cause cell cycle disorders, which results in unlimited cell proliferation [2,3]. Therefore, the cyclin D-CDK4/6-Rb signaling pathway plays a critical role in cell cycle regulation, and inhibition of CDK4/6 has become an effective strategy for tumor treatment [4,5]. Currently, five CDK4/6 inhibitors (CDK4/6is), palbociclib, ribociclib, abemaciclib, dalpiciclib, and trilaciclib, have been approved for use in the clinical treatment of cancer (Table 1). Among them, palbociclib, ribociclib, abemaciclib, and dalpiciclib are employed in combination with endocrine therapy for HR-positive (HR+)/HER2-negative (HER2-) locally advanced or metastatic breast cancer [6], while trilaciclib is utilized as preventive therapy prior to chemotherapy in patients with extensive-stage small-cell lung cancer (ES-SCLC) to reduce the incidence of bone marrow suppression [7].

In recent years, an increasing number of studies have revealed that CDK4/6 kinases regulate a much more extensive group of cellular functions than previously expected. Consequently, in addition to inhibiting tumor cell proliferation, CDK4/6is have also been found to affect tumor cells and the tumor microenvironment through mechanisms that are in early stages of clarification. For instance, CDK4/6is can modulate energy metabolism and induce autophagy and senescence in cells, and CDK4/6is have also been shown to be involved in tumor angiogenesis. Thus, CDK4/6is have also been shown to influence anti-tumor immunity [8,9,10,11,12].

This review aimed to investigate recent preclinical studies on the role of CDK4/6is in tumor immunity. We found that CDK4/6is have dual effects on immune regulation. Notably, CDK4/6is exert anti-tumor immune effects by enhancing tumor antigen presentation, mediating the senescence phenotype that activates the immune system, inducing immunogenic cell death, enhancing T cell infiltration and activation, and reducing immunosuppressive cells. However, CDK4/6is suppress anti-tumor immunity through aberrant activation of the interferon signaling pathway and increased expression of PD-L1 in tumor cells. Although some previous reviews have focused on CDK4/6is and tumor immunity [13,14,15], none have clearly elucidated in which aspects CDK4/6is specifically exhibit “duality”. Additionally, we performed a detailed examination of the clinical trials that utilized a combination of CDK4/6is and immunotherapy for cancer treatment, discussing the problems with the combined treatment strategy and the associations with the “duality” of CDK4/6is, which have not been found in previous reviews. Clarifying the molecular mechanism by which CDK4/6is regulate immunity and determining how their anti-tumor effects can be enhanced to mitigate adverse events are urgent issues that need to be addressed.

## 2. Role of CDK4/6 in Biology

**Figure 1:**
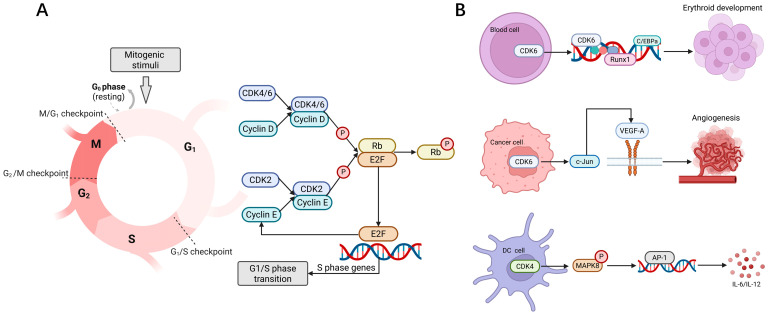
The role of CDK4/6 in biology. (**A**) The role of CDK4/6 in cell cycle regulation. Mitogenic signals stimulate the formation of cyclin D/CDK4/6 complexes, leading to the phosphorylation of Rb protein and partially relieving the inhibition on E2F transcription factor. E2F transcription factor enhances the expression of cyclin E, promoting the formation of cyclin E/CDK2 complexes and further phosphorylating Rb, consequently stimulating the G1/S phase shift. (**B**) Other biological roles of CDK4 and CDK6, apart from regulating the cell cycle. CDK6 promotes erythroid development and angiogenesis. CDK4 triggers the release of IL-6 and IL-12 in DCs.

CDK4/6 plays a particularly crucial and fundamental role in the G1/S transition of the cell cycle [16] (Figure 1A). The Rb protein binds to the transcription factor E2F during the early G1 phase, thereby inhibiting its transcriptional activity [17]. When mitogenic stimuli elevate cyclin D expression, cyclin D forms complexes with CDK4/6, leading to the phosphorylation of Rb. This partially relieves the inhibition on E2F transcription factors, promoting the expression of E2F target genes such as cyclin E [18,19]. The cyclin E-CDK2 complex further phosphorylates Rb, resulting in the complete release of E2F transcription factors [20]. The cell cycle thus formally completes the transition from the G1 to the S phase. CDK4 and CDK6 reportedly share 71% amino acid identity, rendering them highly redundant in cell cycle regulation [21]. However, several studies have indicated biological differences between CDK4 and CDK6 [22,23,24,25,26] (Figure 1B). CDK6 is more highly expressed in blood cells and lymphocytes, where it promotes erythroid development and myeloid differentiation [22,23]. Furthermore, CDK6 regulates vascular endothelial growth factor A (VEGF-A) expression through c-Jun, thereby promoting angiogenesis [24]. In immune regulation, CDK4 phosphorylates mitogen-activated protein kinase 8 (MAPK8) in dendritic cells (DCs), triggering the release of interleukin 6 (IL-6) and interleukin 12 (IL-12) [25]. Additionally, the activity of the cyclin D3-CDK6 complex is essential for maintaining thymocyte numbers [26]. CDK4 and CDK6 also play key roles in the anti-inflammatory response of neutrophils [27]. These findings suggest that, in addition to jointly facilitating cell cycle progression, CDK4/6 may possess additional cell cycle-independent functions, providing potential opportunities for tumor immune regulation through CDK4/6is.

## 3. Activating Anti-Tumor Immunity via CDK4/6is

### 3.1. Enhancing Antigen Processing and Presentation in Tumor Cells

**Figure 2:**
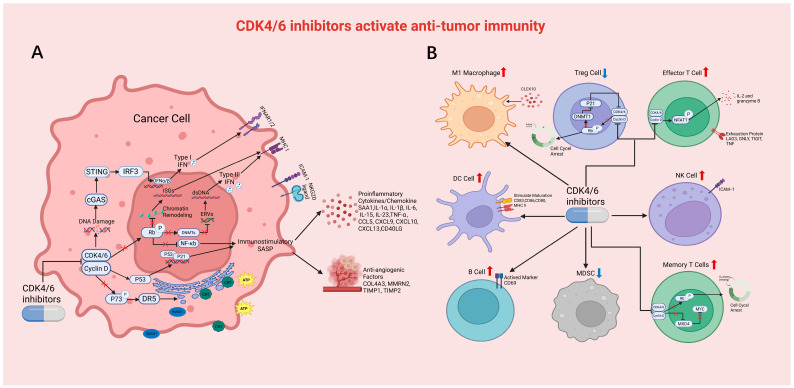
CDK4/6is activate anti-tumor immunity. (**A**) The left half of the picture shows the mechanism by which CDK4/6is act on tumor cells to activate anti-tumor immune. CDK4/6is activate IFN response and upregulate the expression of MHC-I. CDK4/6is promote the occurrence of cellular senescence. The SASP that induces anti-tumor effects mainly consists of some cytokines, chemokines and SASP-dependent vascular remodeling effects. CDK4/6is induce ICD in cells. (**B**) The right half of the picture shows the mechanism by which CDK4/6is act on immune cells to activate anti-tumor immune. CDK4/6is increase the proportion of DCs, M1 ratio and B cell subsets in the immune microenvironment. CDK4/6is inhibit Treg cells and reduce the infiltration of MDSC. CDK4/6is enhance T cell activation and reduce exhaustion. CDK4/6is upregulate NK cell. CDK4/6is promote memory T cells formation.

Interferon genes and MHC molecules are key elements in the antigen presentation of tumor cells. Members of the interferon family induce the expression of proteasomes, generating mature antigen peptides that are loaded onto MHC complexes and presented on the surface of tumor cells, where they are recognized by the T cell receptors of CD8^+^ T cells, triggering the immune response [28]. Previous studies have demonstrated that CDK4/6is, such as palbociclib or abemaciclib, can enhance antigen presentation by improving the expression of interferons or MHC molecules [29]. In a murine model of breast cancer, abemaciclib enhance the expression of endogenous retrovirus (ERV) genes by inhibiting DNA methyltransferases (DNMTs), suppressing double-stranded RNA, and ultimately inducing the production of type III interferons and upregulating MHC-I expression [30]. Another study reported that abemaciclib upregulates Rb-dependent activator protein 1 (AP-1), which in turn, induces chromatin remodeling and interferon-stimulated genes (ISGs) upregulation, ultimately increasing antigen presentation [31] (Figure 2A). In a model of ovarian cancer, palbociclib was also shown to increase the secretion of interferons and ISGs [32]. In addition to the mechanisms of action mediated by inhibiting Rb phosphorylation, similar effects also exist in some Rb-deficient tumors. A previous study found that palbociclib induce endogenous DNA damage in vivo, which activates the cGAS-STING pathway and leads to a type I interferon response [33] (Figure 2A). Moreover, palbociclib promotes the STING-TBK1 pathway in Rb-deficient prostate cancer cells by dephosphorylating TBK1 [34]. Therefore, CDK4/6is enhance antigen presentation through both Rb-dependent and Rb-independent pathways, providing chance for the application of CDK4/6is in Rb-deficient tumors. In addition, in a RAS mutation model, palbociclib combined with trametinib was shown to simultaneously induce G1 phase cell cycle arrest and activate the interferon pathway [35]. A co-delivery system loaded with CDK4/6is and poly ADP-ribose polymerase (PARP) inhibitors was designed to enhance antigen presentation by activating the type I interferon signaling pathway [36].

### 3.2. Mediating the Release of the Immunostimulatory Senescence-Associated Secretory Phenotype by Tumor Cells

Chemotherapy, radiotherapy, and targeted therapeutic drugs, including CDK4/6is, can all induce tumor cell senescence [37,38]. One of the most prominent features of senescent cells is the senescence-associated secretory phenotype (SASP) [10]. Previous studies have reported that the types and functions of the SASP secreted by senescent cells can change depending on various factors, such as cell type and stimulation [10,39].

The SASP that induces anti-tumor effects primarily consists of some cytokines and chemokines, which activate CD8^+^ T cells, DCs, and NK cells in the tumor microenvironment [40,41]. In a breast cancer model, palbociclib can recruit and activate neutrophils by releasing inflammatory factors, such as IL-8 and serum amyloid A1 [42]. In Hepa1-6 cells, palbociclib significantly upregulated the expression of IL-1α, IL-1β, IL-6, IL-15, IL-23, IFN-g, and TNF-α [43]. Abemaciclib enhanced the chemotaxis of B cells and CD8^+^ T cells via a series of chemokines, including IL2, IFN-γ, CCL5, CXCL9, CXCL10, CXCL13, and CD40LG in a murine ovarian cancer model [44]. In addition to acting on immune cells, CDK4/6is can also exert SASP-dependent vascular remodeling effects. Palbociclib was shown to significantly upregulate the expression of anti-angiogenic factors, such as COL4A3, MMRN2, TIMP1, and TIMP2 in senescent breast cancer cells [45] (Figure 2A).

Regarding the mechanisms of activating anti-tumor immunity through senescence-mediated SASP release, current studies have suggested relying on the p53/p21 and pRb/NF-κb signaling pathways. A previous study reported that abemaciclib enhances the binding of p53 to the promoter region of the target gene CDKN1A (p21), thereby promoting the occurrence of cellular senescence in mice and breast cancer patients [46]. Palbociclib induces Rb-mediated senescence and NF-κB-driven pro-inflammatory SASP in tumor cells, including TNF-α and IL-15, thereby leading to NK cell infiltration in lung cancer mouse model [37,47] (Figure 2A). Furthermore, combining palbociclib or ribociclib with lipid nanoparticles and TLR4 agonists can also lead to the synergistic induction of pro-inflammatory SASP factors, activating anti-tumor immunity [48,49]. Palbociclib combined with MEK inhibitors was demonstrated to lead to tumor cell senescence by expressing activated ligands for NK cells, such as the NKG2D ligands and intercellular adhesion molecule 1 (ICAM1), that drive the recruitment of NK cells (CCL2, CCL4, CCL5, CXCL10) and activate NK cells (IL-15, IL-18, TNF-α) in lung cancer models [47]. CDK4/6is can also effectively induce senescence in hepatic tumor-initiating cells, not only promoting the high expression of SASP factors, CCL2 and CXCL10, which leads to an increase in M1 macrophage infiltration, but also significantly increasing the expression of TNF-α secreted by activated macrophages [50]. CDK4/6is mediate tumor cell senescence to release immunostimulatory SASP, achieving the connection between tumor cells and immune cells in the microenvironment, while inhibiting angiogenesis.

### 3.3. Inducing Tumor Cells to Undergo Immunogenic Cell Death

Immunogenic cell death (ICD) triggers a strong immune response by recruiting and activating antigen-presenting cells [51]. CDK4/6is may potentially act as an ICD inducer, both enhancing anti-tumor immunity and generating a long-lasting anti-proliferative protective immune response. Researchers have created a simple and universal nanoparticle platform loaded with palbociclib, which served as an effective inducer of ICD in breast cancer mice models receiving immunotherapy and avoided the adverse reactions of systemic use of CDK4/6is [52]. In preclinical animal models of glioblastoma, the combination therapy of abemaciclib and oncolytic virus VSVD51 significantly suppressed tumor growth and activated ICD in tumor cells [53]. Currently, the primary mechanisms by which CDK4/6is modulate ICD include the dephosphorylation of p73, which transcriptionally activates death receptor 5 (DR5) and promotes ICD [54] (Figure 2A).

Due to the limitations of CDK4/6is in triple-negative breast cancer (TNBC), a CDK4/6i polyamino acid nanoparticle formulation, named NG2000/ABE, was developed; this formulation effectively enhanced the efficacy of abemaciclib by inducing ICD in vitro and in vivo [55,56]. Another study found that the combination of ribociclib and PI3Kα inhibitors can also induce ICD in TNBC [57]. CDK4/6is can effectively enhance immunogenicity depending on the fact of CDK4/6is inducing ICD in tumor cells. The combination of nanomedicine and physical therapy will probably become an innovative research direction in the future.

### 3.4. Increasing T Cell Infiltration and Activation

It is recognized that CDK4/6is can induce tumor cell cycle arrest. However, previous studies have found that they have limited effects on T cell proliferation [58,59], especially abemaciclib, which acts as a more effective CDK4 inhibitor and has a relatively small impact on CDK6, a major kinase in bone marrow progenitor cells [60]. Consequently, this is theorized to be the reason for the lower incidence rate of neutropenia in abemaciclib clinical trials, as well as the no observed leukopenia rates [61]. It may also partly explain the limited inhibition of T cell proliferation observed during in vitro experiments and the increased T cell infiltration in tumors observed in preclinical studies.

CDK4/6is improve T cell infiltration and activation by modulating the levels of various chemokines [17,62]. Abemaciclib promotes the expression of CCL4 in ovarian cancer animal models by inhibiting SCD1, thereby leading to the infiltration of CD8^+^ effector T cells into the tumor [63]. Previous research has shown that after treatment with abemaciclib alone or in combination with an anti-PD-1 antibody in preclinical models of brain metastasis, the levels of anti-inflammatory cytokines, such as LIF, IL-6, and IL-11, increase, while the concentrations of TH1 inhibitory cytokines, such as IL-4, IL-10 and TGF-β, decrease, which increases effector T cell infiltration [64]. Abemaciclib has also been shown to enhance the activity of nuclear factor of activated T cell 1 (NFAT1), increase the transcriptional regulation of IL-2 and granzyme B, and exhibit an enhanced effect on T cells in murine breast tumor models [58] (Figure 2B). Enhancing T cell activation by removing the inhibition on NFAT family proteins and their target genes, thereby enhancing the anti-tumor effect in vivo, has also been confirmed in palbociclib and trilaciclib [65]. After the use of CDK4/6is, the expression of exhaustion genes in T cells, including LAG3, GNLY, TIGIT, and TNF, was significantly reduced [12,66,67] (Figure 2B).

Additionally, CDK4/6is have been reported to influence the activation of effector T cells by acting on tumor cells. Palbociclib enhances the transcription of ICAM1 by inhibiting the phosphorylation of Rb, ultimately leading to the activation of CD8^+^ T cells in LKB1-deficient lung cancer animal model [68]. CDK4/6is, even in Rb-deficient tumors, have been shown to cause double-stranded DNA damage and activate the STING signaling pathway, regulating type I interferon-induced activation and increasing CD8^+^ T cell infiltration [33,36,69]. Given the varying degrees of inhibition on CDK4 and CDK6, more research is needed to confirm the effects of CDK4/6is on T cells, not limited to different types of CDK4/6is.

### 3.5. Decreasing Immunosuppressive Cells

CDK6 is expressed at higher levels in regulatory T cells (Treg cells) as compared to other T cell subtypes, which may explain the elevated sensitivity of Treg cells to CDK4/6is [70]. Abemaciclib not only directly induce cell cycle arrest in Treg cells, leading to a decrease in their proliferation, but have also been found to directly inhibit Treg cells by reducing the level of DNA methyltransferase 1 (DNMT1) and upregulating p21^CIP1^ [30] (Figure 2B). In the dMMR mouse tumor model, the number of Treg cells in circulation and in the spleen was significantly decreased in the preventive abemaciclib treatment group [71,72]. Furthermore, both abemaciclib monotherapy and combination therapy with an anti-PD-1 antibody have been reported to significantly reduce the level of Treg infiltration in intracranial tumors and subcutaneous breast cancer tumors in the 4T1 breast cancer brain metastasis mouse model, and similar results were observed in the spleen and peripheral blood [64,73]. Because the inhibitory effect of palbociclib on Treg cells counteracts the activation effect of IL-2 on Treg cells, combination therapy has been used to effectively enhance the anti-tumor effect by improving the immune microenvironment of colorectal cancer [74]. Researchers have generated a nano-delivery system of proteolysis-targeting chimeras (PROTACs) containing palbociclib in combination with an anti-PD-1 antibody and found significant inhibition of Treg cells, as well as an enhanced effect of immune checkpoint blockade in CT26 tumor model [75]. Another study employed prodrug liposomal nanocarriers that integrate palbociclib, obtaining results similar to a previous study using a mouse model of colon cancer [76].

In addition to Treg, studies have found that CDK4/6is also have an effect on myeloid cells. It has been demonstrated that in HR+ breast cancer patients, the level of monocytic and polymorphonuclear myeloid-derived suppressor cells (MDSCs) in peripheral blood are reduced after treatment with different CDK4/6is [77]. In the 4T1 mouse breast cancer model, the proportion of PMN-MDSCs/neutrophils, monocyte-type myeloid-derived suppressor cells (M-MDSCs), and monocytes (CD11b^+^Ly6G^−^Ly6C^+^) in subcutaneous tumors treated with abemaciclib or abemaciclib combined with an anti-PD-1 antibody was lower compared to treatment using only an anti-PD-1 antibody [73].

### 3.6. Enhancing the Infiltration and Function of Antigen-Presenting Cells

As mentioned earlier, palbociclib can induce senescence and damage-associated molecular patterns (DAMPs), which in turn can stimulate the maturation of DCs. Studies have found in mice models that senescent cells enhance the ability of DCs to acquire and present antigens, and this effect is superior to that of ICD, which has been verified in patient-derived tumor cells [40]. Palbociclib not only increased the proportion of dendritic cells but also increased the expression level of CD83 on the DCs, which was also verified in the peripheral blood samples from breast cancer patients [78] (Figure 2B). Abemaciclib induces antigen presentation in MCF-7 cells [58]. Macrophages and B cells also play crucial roles in anti-tumor immune responses by presenting antigens. In dMMR mice, after abemaciclib treatment, the expression of Csf2 relative to Csf1, which is associated with the M1-like phenotype, showed an upward trend, indicating that abemaciclib monotherapy led to M1 polarization [72]. The combination of XVir-N-31 and ribociclib was shown to induce an increase in the M1/M2 ratio, which was primarily driven by upregulation of CXCL10 in vivo tumor xenograft models [79] (Figure 2B). In preclinical studies of ID8 murine ovarian cancer model, the proportion of B cell subsets after abemaciclib monotherapy in the immune microenvironment and the marker CD69 representing their activation were reported to both be increased (Figure 2B). At the same time, it was found that the level of IL-10 affecting B cell secretion was decreased [44]. Malignant peripheral nerve sheath tumors (MPNSTs) exhibit a significant increase in B cell/plasma cell infiltration along with tumor shrinkage after CDK4/6-MEK inhibition [80].

### 3.7. Inducing T Cell Memory

CDK4/6is also appear to have the ability to shift T cells towards a central memory T cell phenotype, which may make the anti-tumor immune response more resilient. The results of single-cell RNA sequencing on breast cancer and ovarian cancer patients treated with ribociclib found an increase in memory T cells. Further experiments using mouse models demonstrated that enhancement of anti-tumor memory by ribociclib improved the efficacy of subsequent PD-1 blockade therapy [81]. The most likely possible mechanism for this is ribociclib upregulating MXD4 (a negative regulator of MYC) in CD8^+^ T cells, with the inhibition of Myc leading to the formation of memory T cells [82] (Figure 2B). Other studies have found that palbociclib promote memory formation through RB-mediated G1 arrest [83]. Abemaciclib plus an anti-PD-1 antibody significantly increased infiltration of CD45^+^CD3^+^ total T cells, CD3^+^CD8^+^ cytotoxic T cells, and CD44^high^CD62L^low^ memory/effector T cells in a C57BL/6J mouse model [68]. Although current studies on CDK4/6is inducing memory T cells are still relatively scarce, such mechanisms shed new light on the application of CDK4/6is in clinical tumor immunotherapy.

### 3.8. Enhancing NK Cell Surveillance

Palbociclib upregulates the transcription of ICAM-1 by inhibiting the phosphorylation of RB in LKB1-deficient lung cancer animal model, which is essential for NK cell surveillance [68]. Additionally, the activation of ICAM-1 can also be achieved through CDK4/6i-mediated cellular senescence and the release of the SASP [48,57] (Figure 2B).

## 4. Suppression of Anti-Tumor Immunity via CDK4/6is

### 4.1. Interferon Pathway Abnormal Activation

**Figure 3:**
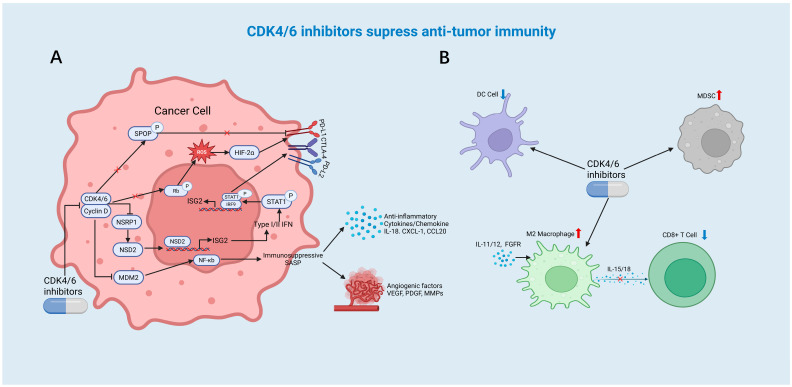
CDK4/6is suppress anti-tumor immunity. (**A**) The left half of the picture shows the mechanism by which CDK4/6is act on tumor cells to suppress anti-tumor immune. CDK4/6is activate the IFN signaling pathway. CDK4/6is lead to the release of immunosuppressive SASP by mediating cellular senescence. CDK4/6is increase the level of PD-L1 on tumor cells. (**B**) The right half of the picture shows the mechanisms by which CDK4/6is act on immune cells to suppress anti-tumor immune. CDK4/6is stimulate the differentiation of M2 macrophages and weakened the IL-15/18 signaling interaction between M2 macrophages and CD8^+^ T cells. CDK4/6is decrease the number of DCs and increase the infiltration of MDSCs.

As mentioned earlier, interferon signaling is a central manner in which tumor cells achieve antigen presentation. However, a large number of studies have shown that the initial exposure of interferon promotes antigen presentation and T cell activation, while the continuous activation of interferon signaling can lead to an immunosuppressive microenvironment by increasing the infiltration of Treg cells and the expression of immune checkpoints [84,85]. Compared to patients with short-term treatment or patients not treated with CDK4/6is for breast cancer, patients who developed acquired resistance to palbociclib exhibited significant activation of the interferon/STAT1 signaling pathway, which induced the expression of immune checkpoints, such as PD-L1, PD-L2, and CTLA-4, on the surface of tumor cells and stromal cells, thereby leading to immunosuppression [86]. In addition, the expression of NSRP1 was downregulated in palbociclib-resistant breast cancer cells. Mediating the alternative splicing of NSD2 mRNA further activated the interferon signaling pathway [87] (Figure 3A). Another study demonstrated that in patients resistant to palbociclib, stimulatory genes, such as ICOS, CD70, and CD27, were suppressed [88]. Therefore, balanced interferon signaling is a significant influencing factor in regulating tumor immunity.

### 4.2. Mediating the Release of the Immunosuppressive SASP by Tumor Cells

CDK4/6is-induced cellular senescence plays a dual role in tumor immunity [10]. Senescent tumor cells can also secrete an immunosuppressive SASP, including interleukin-18, CXCL-1, and CCL-20, by CDK4/6is, increasing the infiltration of MDSCs and leading to an immunosuppressive microenvironment [89,90]. Palbociclib has been proven to induce SASP, which leads to the upregulation of CCL2. CCL2 attracts Tregs to the tumor microenvironment, where they exert immunosuppressive effects. The use of a CCL2 inhibitor, pirfenidone, can enhance the anti-tumor effect of palbociclib [91]. In addition, the immunosuppressive function of SASP is also reflected in promoting angiogenesis. Palbociclib and trametinib induced a SASP rich in angiogenic factors, including VEGF, PDGF, and matrix metalloproteinases (MMPs), effectively increasing angiogenesis in Kras-mutated pancreatic ductal adenocarcinoma (PDAC) [92,93]. At present, there are limited studies regarding the mechanism of a SASP-induced immunosuppressive microenvironment. Other research has shown that palbociclib lead to the release of an immunosuppressive SASP by modulating the downregulation of Mdm2 expression and activating the NF-κB signaling pathway, thereby creating an immunosuppressive microenvironment in preclinical models of melanoma [94] (Figure 3A). Understanding the SASPs triggered by various CDK4/6is will help to fully utilize the anti-tumor and immune-stimulating properties of senescence to treat tumors or develop better combined treatment strategies.

### 4.3. Enhancing the Expression of PD-L1 in Tumor Cells Mediates Immune Escape

A study based on 85 patients in 2019 found that the copy number of CDK4 was negatively correlated with the efficacy of anti-PD-1 antibodies, thus initiating the research investigation into CDK4/6is and immune checkpoint proteins [95]. In hepatocellular carcinoma, even malignant peripheral nerve sheath tumors (MPNSTs), CDK4/6is cause tumors to be more sensitive to PD-L1 blockade therapy by increasing the expression of PD-L1 [43,80]. The PARP inhibitor talazoparib, in combination with palbociclib and nano-PROTACs for palbociclib, has been shown to increase the expression of PD-L1 in tumor cells and synergistically enhance the effect of immune checkpoint blockade in immunocompetent mouse colorectal cancer models [75,96].

The mechanism underlying the enhancement of PD-L1 expression is that palbociclib can induce an increase in ROS, specifically H_2_O_2_, leading to enhanced expression of HIF-2α, which subsequently upregulates the PD-L1 [97]. A previous study showed that impeding the cyclin D-CDK4-dependent phosphorylation of speckle-type POZ protein (SPOP) promotes the degradation of SPOP by the activator of the anaphase-promoting complex FZR1, thereby increasing the protein level of PD-L1 [98] (Figure 3A). After treatment with palbociclib, genes in the NF-κB pathway, including PD-L1, were upregulated [99,100]. Abemaciclib and palbociclib, as functional p16 mimics, have been shown to induce cellular senescence and thereby increase the stability of PD-L1 protein, halting its protein degradation [101]. Given that CDK4/6is increase PD-L1 expression, the combination of CDK4/6is with anti-PD-L1 monoclonal antibodies becomes an ideal choice. Additionally, whether CDK4/6is also regulate other immune checkpoint inhibitors or immunosuppressive proteins deserves further exploration.

### 4.4. Inducing Unexpected Alterations in Immune Cells Within the Tumor Microenvironment

The effects of CDK4/6is on antigen-presenting cells and MDSCs also vary, and no dual effects on other immune cells have been discovered until now. In patients with early luminal B-type breast cancer, the expression of features related to antigen-presenting cells decreased after 14 days of treatment with ribociclib [102]. In cells that developed resistance to ribociclib treatment, single-cell sequencing results indicated that the expression of cytokines and growth factors, such as IL-11/12 and FGFR, was upregulated, which stimulated the differentiation of M2-polarized macrophages and weakened the IL-15/18 signaling interaction with CD8^+^ T cells, resulting in an immunosuppressive effect [103] (Figure 3B). It was also discovered that palbociclib might have an adverse effect on the differentiation of bone marrow progenitor cells into DCs in muring breast cancer models, but the specific mechanism remains unclear [104]. Another study, which focused on patients with advanced breast cancer treated with different CDK4/6is, also confirmed through single-cell sequencing technology that the number of antigen-presenting cells in samples from drug-resistant patients was significantly reduced [66].

In preclinical studies of melanoma, palbociclib treatment has been shown to mediate cellular senescence and increase the infiltration of granulocyte MDSCs [94]. Additionally, although the combined inhibition of BRAF, MEK, and CDK4/6 has shown promising anti-tumor effects, flow cytometry analysis has indicated that the massive infiltration of tumor-infiltrating myeloid cells may have an adverse impact on anti-tumor immunity in melanoma [105]. With regard to HR+/HER2+ breast cancer transgenic mouse model, the combination therapy of anti-HER2 antibodies and palbociclib has failed to achieve the expected therapeutic effect. Through high-throughput single-cell analysis, a previous study found that a unique type of immunosuppressive immature myeloid cell (IMC) had significantly infiltrated the tumor microenvironment, which led to treatment failure [106]. Further work is required to elucidate how CDK4/6is affect antigen-presenting cells and their interactions with MDSCs.

## 5. Clinical Trials for the Combination of CDK4/6is and Immunotherapy

From the above preclinical studies, we can conclude that CDK4/6is, in addition to inducing cell cycle arrest, also have unexpected immunomodulatory effects [30]. Given that CDK4/6is can increase the expression of PD-L1 in tumor cells, a large number of studies have investigated the anti-tumor and immune regulatory effects of CDK4/6i sin combination with anti-PD-1/PD-L1 antibodies. As expected, CDK4/6is not only significantly improved the efficacy of anti-PD-1/PD-L1 antibodies in animal models but also reduced immunosuppressive cells [73,75] and increased the infiltration of cytotoxic T cells and memory T cells [69]. In recent years, a large number of clinical studies have focused on investigating CDK4/6is combined with immunotherapy. However, as mentioned above, CDK4/6is exhibit dual immunomodulatory effects, and the results of these clinical trials are worthy of a thorough discussion.

The approved CDK4/6is include palbociclib, ribociclib, abemaciclib, dalpiciclib, and trilaciclib. Combination therapy utilizing various CDK4/6is and immunotherapy has shown that the current investigations are still at the phase I/II clinical trial level [107,108,109] (Supplementary Table S1). The majority of the combined immunotherapy options were anti-PD-1/PD-L1 antibodies. The tumor types explored have included lung cancer, breast cancer, squamous cell carcinoma of the head and neck, liposarcoma, and some primary or metastatic intracranial tumors. We summarize several key clinical research findings in Table 2. A phase I/II trial has tested the safety and efficacy of palbociclib, pembrolizumab, and letrozole as a first-line treatment for HR+/HER2− advanced breast cancer patients [110]. The results of this study confirmed that pembrolizumab combined with palbociclib and letrozole as a first-line treatment had an overall PFS comparable to 24.8 months in the PALOMA-2 trial [111] and a complete response rate of 31%. In terms of safety, grade 3/4 adverse events were mainly hematological toxicity and liver function abnormalities. Another exciting outcome was the PACE research, which focused on patients with advanced breast cancer who experienced disease progression after receiving CDK4/6i + AI [112]. Fulvestrant plus palbociclib and avelumab prolonged PFS (the median PFS was 8.1 months (hazard ratio [HR] v F 0.75 [90% CI, 0.50–1.12]; *p* = 0.23)) compared to fulvestrant (the median PFS was 4.8 months) or fulvestrant plus palbociclib (the median PFS was 4.6 months (HR, 1.11 [90% CI, 0.79–1.55]; *p* = 0.62)), although this was not statistically significant. Notably, among patients with endocrine-sensitive diseases, the median progression-free survival for fulvestrant was 5.7 months (90% CI: 2.3–9.9), for the fulvestrant plus palbociclib was 4.6 months (90% CI: 3.5–9), and for the fulvestrant plus palbociclib and avelumab was 8.5 months (90% CI: 5.7–19.0). A total of 84 (38.9%) experienced a grade 3/4 treatment-related adverse event (TRAE). No cases of pneumonia or interstitial lung disease were observed. However, the results of abemaciclib combined with immunotherapy were somewhat unsatisfactory [113,114,115]. The NCT02779751 study investigated the application of abemaciclib combined with pembrolizumab in NSCLC and breast cancer. The results of the NSCLC part published in 2021 indicated that in PD-L1-positive, KRAS-mutated, previously untreated non-squamous stage IV NSCLC patients [113], the incidence of transaminase elevation and pneumonia (n = 8 each; 32.0%) within combined therapy was higher than that previously reported for the use of these two drugs alone, and its efficacy (the median PFS was 7.6 months (95% CI: 1.6–not estimable), the median OS was 27.8 months (95% CI: 9.9–not estimable)) was basically equivalent to that of single-agent immunotherapy [116,117]. The breast cancer portion of the study led by Professor Hope S. Rugo [115] was divided into two cohorts: treatment-naïve (cohort 1) and pretreated (cohort 2). The results indicated that although the median PFS (8.9 months (95% CI: 3.9–11.1)) and OS (26.3 months (95% CI: 20.0–31.0)) in the cohort that received abemaciclib plus pembrolizumab were higher compared to the MONARCH 1 [118] and KEYNOTE-028 [119] studies, the incidence of interstitial lung disease/pneumonia (grade ≥ 3 ILD/pneumonitis = 7.7%) and severe transaminase elevations (grade ≥ 3 ALT increased = 42.3%, grade ≥ 3 AST increased = 34.6%) was high regardless of whether anastrozole was used. Apart from the studies in advanced tumors, the examination of combining palbociclib with nivolumab in neoadjuvant treatment for breast cancer also ended in failure due to safety issues, such as a relatively high incidence of grade 3–4 hepatotoxicity (n = 6, 66.7%) [120]. The results of ribociclib combined with an anti-PD-1 monoclonal antibody also reported intolerable safety issues [121]. Trilaciclib, a CDK4/6 inhibitor approved for the indication of reducing the risk of myelosuppression, has not been found to have anti-tumor effects in previous studies. However, the results of a recent phase II clinical study demonstrated that adding trilaciclib prior to the gemcitabine plus carboplatin (GCb) combination therapy in patients with advanced TNBC significantly prolonged the ORR and OS. Moreover, subgroup analyses, including the enhancement of T cell activation, suggested that the efficacy outcomes may be mediated through immune mechanisms [122]. Some studies have shown that trilaciclib enhances anti-tumor immunity by differentially inhibiting cytotoxic T cells and Treg cells [17,65,123]. However, there are no clinical trials exploring the combination of trilaciclib and immunotherapy at present.

From the clinical trials of CDK4/6is combined with immune checkpoint inhibitors (ICI), only the PACE study reported prolonged PFS with fulvestrant, palbociclib and avelumab in patients whose disease progressed after CDK4/6is + AI, although this improvement did not reach statistical significance and the regimen was relatively well tolerated [112]. No other trial has demonstrated marked therapeutic benefit, irrespective of the CDK4/6is or ICI employed. Notably, the primary reason for failure of the CDK4/6is combination therapy with ICI regimens is attributed to severe drug-related adverse events, with liver toxicity, pneumonia/interstitial pneumonia, and bone marrow suppression being prominent [113,115,120]. At present, only one study has conducted a relatively in-depth analysis of the liver toxicity of CDK4/6is combined with ICI [124]. This multi-cohort phase II study evaluated the efficacy and safety of nivolumab combined with abemaciclib and fulvestrant or letrozole as a first-line/second-line treatment for HR+/HER2− advanced breast cancer. though it was prematurely terminated due to a high incidence of liver toxicity. A total of 17 patients were enrolled in the trial (12 in the fulvestrant cohort and 5 in the letrozole cohort). ORRs were 54.5% (6/11) in the fulvestrant and 40.0% (2/5) in letrozole cohorts, respectively. At the time of safety review, 6 out of 17 patients experienced grade ≥ 3 hepatotoxicity, which led to treatment termination. The most frequent non-hematological grade ≥ 3 TEAE was ALT elevation (41.6% in the fulvestrant cohort and 40.0% in letrozole cohorts). Biopsies of liver specimens from three patients confirmed that CD3^+^ and CD8^+^ lymphocytes were predominant. The ratio of CD4/CD8 indicated that hepatotoxicity was likely immune-related. Additionally, substantially elevated serum cytokines (such as sCD30/TNFRSF8, Thymic stromal lymphopoietin (TSLP), IL-11, IL-12 (p40), pentraxin-3, sTNF-R2, sTNF-R1, IL-34 and interferon-β) and reduced peripheral effector Treg cells were detected, which also supported this inference. Further results indicated that the inhibition of PD-1-positive regulatory T cells by abemaciclib may be the cause of the serious adverse events. This study was also the first to demonstrate, at the pathological and immunological levels, that CDK4/6is enhance the immune response mediated by ICI in a clinical trial. However, the enhancement of this immune response led to uncontrollable adverse events, and as a result, clinical trials failed to achieve the expected therapeutic effect. At present, there are some ongoing clinical trials exploring the combination of CDK4/6is and immunotherapy (Table 3). Considering the dual immune regulatory effect of CDK4/6is, a large number of studies are still needed to further clarify the impact of various CDK4/6is on the immune microenvironment in clinical practice. Additionally, further studies should be aimed at not only understanding how to enhance the anti-tumor effect on the basis of improving adverse events but also at exploring combination therapy with immune therapeutic agents other than ICIs.

In addition to CDK4/6is, inhibiting other cyclin-dependent kinases can also affect tumor immunity. CDK2 inhibitors enhance anti-tumor immunity by regulating the IFN signaling pathway [125,126] and improving the tumor immune microenvironment [127]. CDK2/7/9 inhibitors induce tumor cell debris, enhancing the therapeutic effect of anti-PD-L1 monoclonal antibodies [128]. The highly selective CDK7 inhibitor activates CD8^+^ T cells through the secretion of some pro-inflammatory cytokines [129] and inhibits PD-L1 expression through the p38α-MYC axis [130]. CDK12/13 inhibitors induce ICD to enhance anti-tumor immunity [131]. However, the combinations of these cyclin-dependent kinase inhibitors with immunotherapy are currently in the preclinical studies.

## 6. Raising Discussions and Perspectives

This review presents a comprehensive analysis of the role of CDK4/6is in tumor immunity. Preclinical evidence indicates that CDK4/6is exert anti-tumor effects by acting on malignant cells and on diverse immune cells within the microenvironment. The mechanisms identified include enhanced antigen presentation via the interferon signaling pathway [29,30,31,32,33,34], the release of immune-stimulatory SASP from senescent cells [42,43,44,45,46,47], the induction of ICD in tumor cells [52,53,54,55,56,57], increased T cell infiltration [62,63,64,65,66,67,68,69], the reduction In Treg cells [30,71,72,73,74,75,76], and the promotion of a memory T cell phenotype [68,82,83,84]. To date, no substantial differences have been demonstrated among individual CDK4/6is in their activation of anti-tumor immunity. No preclinical studies have directly compared the effects of different CDK4/6is on a given immune signaling pathway, nor has any specific action unique to a single inhibitor been identified. One exception is that preclinical work focused on enhancing T cell infiltration and activation has predominantly implicated abemaciclib [58,63,64], a finding that may relate to its relatively smaller effect on CDK6 [60]. This study also provides a detailed summary of how CDK4/6is suppress anti-tumor immunity. It describes abnormal activation of interferon signaling [84,85,86,87,88], induction of immunosuppressive SASP from senescent cells [89,90,91,92,93,94], upregulation of PD-L1 on tumor cells [97,98,99,100,101], and distinct effects on two antigen-presenting cell subpopulations and on MDSCs [102,103,104,105,106]. Together, these findings substantiate the “dual role” of CDK4/6is in tumor immunity. Inflammatory mediators such as interferon-γ (IFN-γ) are well established drivers of PD-L1 expression [132,133]. The IFN-γ–IFNGR1/2–JAK1/2–STAT1–IRF1 axis is central to anti-tumor immunity, and dysregulation of this pathway can perturb PD-L1 levels and alter sensitivity to immunotherapy [134,135,136]. Although CDK4/6is have been reported to increase PD-L1 on tumor cells, this effect has not been clearly linked to IFN-γ signaling; nonetheless, prolonged IFN-γ exposure, which can induce a T cell exhaustion phenotype, remains a plausible contributor to PD-L1 upregulation [137]. Aberrant activation of IFN-γ signaling is also among the mechanisms by which CDK4/6is can impair anti-tumor immunity, as noted above. Thus, CDK4/6is-driven PD-L1 elevation exemplifies the drugs’ dualistic effects on tumor immunity. Given the temporally dependent roles of interferon signaling, the divergent impacts of early versus long-term CDK4/6is exposure on the immune microenvironment merit further detailed investigation.

Summarizing the clinical trial results of CDK4/6is in combination with ICI, the adverse reactions related to treatment are remarkable, which has become the main obstacle to the development of such combination therapies. Even in the PACE study, the relatively better safety profile reflected in the results might be attributed to the fact that 40% of the patients started with a reduced dose of palbociclib [112]. This further suggests that reducing the dosage or the duration of CDK4/6is therapy may solve the problem of severe adverse events when combined with immunotherapy. Research has found that low doses of abemaciclib and palbociclib induce the expression of MHC ligands in specific pathways [138], suggesting that reducing the dose of CDK4/6is therapy might be feasible. However, there are currently no clinical trials investigating if the dosage of CDK4/6is therapy can be reduced or how the duration of CDK4/6is therapy can be shortened to help improve drug-related adverse events. Whether the differences in immunotherapy agents combined with CDK4/6is will affect drug-related adverse events remains unknown, as there are no clinical trials comparing the safety events caused by CDK4/6is combined with different immune agents. From the results we have summarized, the adverse events of CDK4/6is combined with anti-PD-L1 monoclonal antibodies are mostly hematological toxicity, while grade ≥ 3–5 transaminase increase or interstitial pneumonia seems to have a lower incidence (Table 2). Higher-grade liver function damage and pneumonia often lead to treatment suspension, withdrawal, or even death of patients. Previous studies have found that in patients with different advanced or metastatic malignant tumors, a higher incidence of adverse events may be unrelated to the type of immunotherapy [139,140,141]. A meta-analysis of perioperative immunotherapy for various solid tumors contained thirty-one trials (14,974 patients). Safety analyses showed that anti-PD-1 monoclonal antibodies seem to have a higher incidence of grade 3–5 immune-related adverse events (irAEs) than anti-PD-L1 monoclonal antibodies. Frequentist and Bayesian analyses yielded consistent results [142]. Therefore, whether choosing CDK4/6is combined with anti-PD-L1 monoclonal antibodies can promote drug-related adverse events requires more exploration. It is also worth noting that the current clinical data have not yet found that the efficacy was related to PD-L1, TILs, or TMB [110,115,121]. Furthermore, immune-modulatory effects of CDK4/6is have also been observed in the clinic. Although immunological measures were not prospectively specified as primary or secondary endpoints in the PALOMA, MONARCH and MONALEESA trials, a post hoc analysis of MONARCH 2 found that a low baseline neutrophil-to-lymphocyte ratio (NLR) and a high baseline absolute lymphocyte count correlated with longer PFS and OS, and that baseline NLR was an independent prognostic factor for these outcomes [143]. In a prospective study of HR+/HER2− metastatic breast cancer treated with a CDK4/6is plus endocrine therapy, circulating Treg cells and myeloid-derived suppressor cells decreased significantly, while CD4^+^T cells and cytotoxic CD8^+^T cells with anti-tumor phenotype increased [144]. A current clinical study (NCT05766410) is comparing the immunomodulatory effects of palbociclib, ribociclib and abemaciclib each combined with letrozole in the neoadjuvant setting for HR+/HER2− early-stage breast cancer; the trial is actively recruiting and its results are eagerly awaited. Overall, most published trials have not incorporated analyses of human blood or tissue to dissect the specific in vivo immunomodulatory mechanisms of CDK4/6is (Table 2). Consequently, clinical evidence clarifying how CDK4/6is activate or suppress anti-tumor immunity remains limited.

## 7. Conclusions

Currently, the application of CDK4/6is in anti-tumor immunotherapy remains a controversial topic. The suppressive anti-tumor immunity caused by CDK4/6is is primarily related to antigen presentation and the mediation of cellular senescence. This partly explains the possible reasons for the general efficacy of CDK4/6is combined with immunotherapy in clinical trials. Additionally, it is also worth noting that the severe adverse events caused by the combination of CDK4/6is and ICI are currently the main obstacle to the application of CDK4/6is in anti-tumor immunotherapy. Surprisingly, some studies have confirmed that these adverse events are immune-related, which provides strong clinical evidence that CDK4/6is can activate the immune system. This also indicates that further research is still needed to help clarify the molecular mechanisms underlying CDK4/6is-regulated immunity, explore how to avoid severe immune-related adverse events, and improve the efficacy of anti-tumor immunotherapy by adjusting the treatment mode of CDK4/6is or the choice of combined immunotherapy agents in the future. In addition, investigating the influencing factors or biomarkers related to their efficacy or safety may also help improve the outcome of CDK4/6is combined with immunotherapy.

## Figures and Tables

**Table 1:** Characteristics of CDK4/6is approved in clinical trials.

Names	Structure	Targets and IC50	Half-Life, h	Approved Year	Conditions	Dosage
Palbociclib	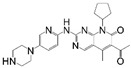	CDK4: 11 nM CDK6: 16 nM	29–33	2015	⮚ HR+/HER2− locally advanced or metastatic breast cancer 1. Combined with aromatase inhibitors as the initial endocrine therapy for postmenopausal women	125 mg po once daily for 21 days, 28 day a cycle
Abemaciclib	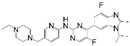	CDK4: 2 nM CDK6: 10 nM	18.3	2016	⮚ HR+/HER2− locally advanced or metastatic breast cancer 1. Combined with aromatase inhibitors as the initial endocrine therapy for postmenopausal women 2. Combined with fulvestrant for patients who have experienced disease progression after previous endocrine therapy ⮚ HR+/HER2− early-stage breast cancer, lymph node-positive, and at high risk of recurrence 1. Combined with endocrine therapy (tamoxifen or aromatase inhibitors) used for the adjuvant treatment	150 mg po bid
Ribociclib	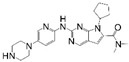	CDK4: 10 nM CDK6: 39 nM	32	2017	⮚ HR+/HER2− locally advanced or metastatic breast cancer 1. Combined with aromatase inhibitors as the initial endocrine therapy for female patients 2. Combined with endocrine therapy and LHRH agonists to treat premenopausal or perimenopausal female patients	600 mg po once daily for 21 days, 28 day a cycle
Trilaciclib	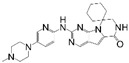	CDK4: 1 nM CDK6: 4 nM	14	2021	⮚ Extensive-stage small-cell lung cancer who have not received systemic chemotherapy before 1. Before treatment with a platinum-based drug combined with etoposide regimen to reduce the incidence of chemotherapy-induced myelosuppression	240 mg/m^2^, 30 min intravenous infusion, completed within 4 h prior to the chemotherapy
Dalpiciclib	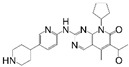	CDK4: 12 nM CDK6: 10 nM	34–37	2022	⮚ HR+/HER2− locally advanced or metastatic breast cancer 1. Combined with aromatase inhibitors as the initial endocrine therapy 2. Combined with fulvestrant for patients who have experienced disease progression after previous endocrine therapy	150 mg po once daily for 21 days, 28 day a cycle

Abbreviation: Hormone Receptor, HR; Human Epidermal Growth Factor Receptor 2, HER2; Luteinizing Hormone-Releasing Hormone, LHRH.

**Table 2:** Summary of key clinical trials on combination of CDK4/6is and immunotherapy.

NCT Number	Study Title	Study Status	Duration of Research	Phases	Conditions	Enrollment	Research Design and Arms	Important Safety Notice	Summary of Effectiveness	Immunomodulatory Effects	Study Results
NCT02778685	Pembrolizumab, endocrine therapy, and palbociclib in treating postmenopausal patients with newly diagnosed metastatic stage IV estrogen receptor-positive breast cancer	Active not recruiting	2016/9/30-	Phase I/II	Metastatic HR+\HER2− breast cancer	20	Pembrolizumab + Letrozole + Palbociclib	G3–4 toxicities were neutropenia (83%), leucopenia (65%), lymphocytopenia (26%), elevated LFTs (17%)	The median PFS and OS were 25.2 m and 36.9 m, respectively	The proportion of type 1 dendritic cells in the circulating dendritic cells increases.	[110]
NCT03147287	Palbociclib after CDK and endocrine therapy (PACE)	Active not recruiting	2017/9/5-	Phase II	Metastatic HR+\HER2− breast cancer, which has progressed on previous CDK4/6i+ET (AI or tamoxifen)	220	Arm A: Fulvestrant (F) Arm B: Fulvestrant + Palbociclib (F + P) Arm C: Fulvestrant + Palbociclib + Avelumab (F + P + A)	The most common G3–4 TRAEs with F + P + A included neutropenia (49.1%), fatigue (5.7%). The most common G3–4 irAEs were increased AST (1.9%) and ALT (1.9%). No cases of pneumonitis or ILD were observed.	The median PFS were 4.8 m (Arm A), 4.6 m (Arm B) and 8.1 m (Arm C)	Not mentioned.	[112]
NCT03498378	Avelumab, cetuximab, and palbociclib in recurrent or metastatic head and neck squamous cell carcinoma	Active not recruiting	2018/6/6-	Phase I	Recurrent/metastatic HNSCC	24	Avelumab + Cetuximab + Palbociclib (75/100/125 mg)	9 patients (75%) experienced G3–4 TRAEs, most of which were hematological toxicities.	3 patients (25%) experienced CR. The median PFS was 6.5 m and the median OS was not reached	Not mentioned.	[109]
NCT02791334	A study of anti-PD-L1 checkpoint antibody (LY3300054) alone and in combination in participants with advanced refractory solid tumors (PACTs)	Completed	2016/6/29–2024/6/27	Phase I	Advanced refractory solid tumors (The study protocol enrolled patients without liver metastases and with normal liver enzyme levels)	81	Arm A: LY3300054 (70/200/700 mg, Q2W) Arm C: LY3300054 (70/200/700 mg, Q2W) + Abemaciclib	7 patients (35%) in Arm C had G ≥ 3 TRAEs: diarrhea, fatigue, anemia, maculopapular rash, neutropenia.	DCRs of Arm C were 75.0% in the 70 mg, 50.0% in the 200 mg and 37.5% in the 700 mg	Increased frequencies in proliferating and activated T cells were not observed in the abemaciclib combination arm.	[114]
NCT02779751	A study of abemaciclib (LY2835219) in participants with non-small-cell lung cancer or breast cancer	Active not Recruiting	2016/11/14-	Phase Ib	Cohort A: Stage IV KRAS-mutant, PD-L1-positive (TPS ≥ 1%) NSCLC; Cohort B: Stage IV squamous NSCLC previously treated by one platinum chemotherapy	25	Abemaciclib + Pembrolizumab	Transaminase elevation (G ≥ 3 = 24%) and pneumonitis (G ≥ 3 = 12%) in cohort 1 were higher than which has previously been reported for either drug alone (One patient experienced a study treatment-related death of pneumonitis in cohort 1)	Cohort A: median PFS and OS were 7.6 m and 27.8 m, respectively, Cohort B: median PFS and OS were 3.3 m and 6.0 m, respectively	Not mentioned.	[113]
NCT02779751	A study of abemaciclib (LY2835219) in participants with non-small-cell lung cancer or breast cancer	Active not Recruiting	2016/11/14-	Phase Ib	Cohort 1: Metastatic HR+\HER2− breast cancer without systemic therapy in the metastasis Cohort 2: Metastatic HR+\HER2− breast cancer treated with ≥1 but ≤2 chemotherapy regimens in the metastasis	54	Arm A (Cohort 1): Abemaciclib + Pembrolizumab + Anastrozole Arm B (Cohort 2): Abemaciclib + Pembrolizumab	Cohort 1, 2 patients died due to TRAEs (both related to ILD). G ≥ 3 TEAEs were 69.2% (ALT increased (42.3%), AST increased (34.6%)). Cohort 2, G ≥ 3 TEAEs were 60.7% (neutropenia (28.6%), AST increased (17.9%)).	Cohort 1: Not reach; Cohort 2: The median PFS and OS were 8.9 m and 26.3 m, respectively	Not mentioned.	[115]
NCT04075604	A study of neoadjuvant nivolumab + palbociclib + anastrozole in post-menopausal women and men with primary breast cancer (CheckMate 7A8)	Completed	2019/10/18–2021/7/27	Phase Ib/II	Primary ER+/HER2− breast cancer	23	Arm A: Abemaciclib + Nivolumab + Anastrozole Arm B: Palbociclib (125 mg, 100 mg) + Nivolumab + Anastrozole	Arm A was closed after enrolling 2 patients due to suggested risk of ILD/pneumonia [115,124]. Arm B was halted with 6 of 21 patients discontinuing treatment due to hepatotoxicity.	pCR was 0 of 9 patients in the palbociclib 125 mg group and 1 of 12 (8.3%) patients in the palbociclib 100 mg group	Not mentioned.	[120]
NCT03294694	Ribociclib + PDR001 in breast cancer and ovarian cancer	Terminated	2017/11/8–2020/10/14	Phase I	Metastatic HR+\HER2− breast cancer Metastatic Ovarian Cancer	33	Arm A: Ribociclib (400/600 mg) + Spartalizumab Arm B: Ribociclib (600 mg) + Spartalizumab + Fulvestrant	Due the high rate of G3–4 hepatotoxicity (7/15) observed in Arm B, the study was closed early after 33 patients enrolled	The addition of spartalizumab had no significant improvement in the efficacy	Ribociclib+ spartalizumab induced a significant increase in circulating CD3^+^ CD45RO^+^ T cells that expressed the activation markers CD38, HLA-DR, and CX3CR1.	[121]
NCT03041311	Carboplatin, Etoposide, and Atezolizumab with or without Trilaciclib (G1T28), a CDK4/6 Inhibitor, in Extensive-Stage SCLC	Terminated	2017/6/29–2020/10/29	Phase II	ES-SCLC	107	Arm A: Trilaciclib prior to chemotherapy and Atezolizumab Arm B: Placebo + chemotherapy and Atezolizumab	One (1.9%) SAE (Grade 2 deep vein thrombosis) was considered related to trilaciclib.	No significant differences in ORR, median PFS and OS	Arm A had a higher ratio of CD8^+^ T cells, activated CD8^+^ T cells to Tregs, and more clonal expansion.	[108]
NCT02978716	Trilaciclib (G1T28), a CDK 4/6 Inhibitor, in Combination with Gemcitabine and Carboplatin in Metastatic Triple-Negative Breast Cancer (mTNBC)	Terminated	2017/2/2–2020/2/28	Phase II	TNBC	102	Arm A: GCb on days 1 and 8 Arm B: Trilaciclib prior to GCb on days 1 and 8 Arm C: Trilaciclib days 1 and 8, and trilaciclib prior to GCb on days 2 and 9	Not mentioned	Median OS was 12.6 m for Arm A, not reached for Arm B (*p* = 0.0016), 17.8 m for Arm C (*p* = 0.0004)	Patients who responded to GCb plus trilaciclib had a higher fraction of newly expanded clones than patients who responded to GCb alone.	[122]

Abbreviation: Hormone Receptor, HR; Grade, G; Liver function tests, LFTs; Progression-free survival, PFS; Overall survival, OS; Endocrine therapy, ET; Aromatase inhibitors, AI; Treatment related adverse event, TRAE; Immune-related adverse events, irAE; Interstitial lung disease, ILD; Head and neck squamous cell carcinoma, HNSCC; Complete response, CR; Disease control rate, DCR; Non-small-cell lung cancer, NSCLC; Estrogen receptor, ER; Treatment emergent adverse event, TEAE; Pathological complete response, pCR; Extensive-Stage small-cell lung cancer, ES-SCLC; Serious adverse event, SAE; Objective response rate, ORR; Triple-Negative Breast Cancer, TNBC; Gemcitabine Carboplatin, GCb.

**Table 3:** Ongoing clinical trials on the combination of CDK4/6is and immunotherapy.

NCT Number	Study Title	Study Status	Conditions	Interventions	Phases	Enrollment
NCT05766410	A Randomized Study Comparing the Immune Modulation Effect of Ribociclib, Palbociclib, and Abemaciclib in ER+/HER2− EBC	Recruiting	Breast Cancer	Palbociclib Ribociclib Abemaciclib Letrozole	Phase II	60
NCT06113809	Palbociclib and Pembrolizumab in Sarcoma	Recruiting	Sarcoma	Palbociclib Pembrolizumab	Phase I	8
NCT05205200	Immune Therapy in HR+/HER2− Metastatic Breast Cancer (ENIGMA)-BCTOP-L-M02	Recruiting	Breast Cancer	SHR-1316 SHR6390 Nab paclitaxel SERD/AI	Phase II	338
NCT06109207	Neoadjuvant Camrelizumab with Dalpiciclib for Resectable Head and Neck Squamous Cell Carcinomas	Recruiting	Head and Neck Squamous Cell Carcinoma	Camrelizumab Dalpiciclib Dalpiciclib	Phase I	6
NCT06654297	Neoadjuvant Camrelizumab with Palbociclib for Resectable Esophageal Squamous Cell Carcinomas	Recruiting	Esophageal Squamous Cell Carcinoma	Camrelizumab Palbociclib	Phase I	6
NCT04360941	PAveMenT: Palbociclib and Avelumab in Metastatic AR+ Triple-Negative Breast Cancer	Recruiting	Breast Cancer	Palbociclib Avelumab	Phase I	45
NCT02896335	Palbociclib and Pembrolizumab in Central Nervous System Metastases	Recruiting	Metastatic Malignant Neoplasm to Brain|	Palbociclib Pembrolizumab	Phase II	45
NCT06364956	Phase Ib/II Trail of Neoadjuvant of Tislelizumab Combined with Palbociclib in Patients with Platinum-refractory Bladder Urothelial Carcinoma	Recruiting	Bladder Cancer	Tislelizumab Palbociclib	Phase I/II	36
NCT04841148	Avelumab or Hydroxychloroquine with or Without Palbociclib to Eliminate Dormant Breast Cancer	Recruiting	Breast Cancer	Hydroxychloroquine Avelumab Palbociclib	Phase II	96

## Supplementary Materials

The following supporting information can be downloaded at: https://www.mdpi.com/article/10.3390/cancers17243997/s1, Table S1: Summary of clinical trials on the combination of CDK4/6i and immunotherapy.

## Abbreviations

CDK4/6: cyclin-dependent kinase 4/6; Rb, retinoblastoma; CDK4/6i, CDK4/6 inhibitors; HR+/HER2−, HR-positive/HER2-negative; ES-SCLC, extensive-stage small-cell lung cancer; CDK2, cycle-dependent kinase 2; VEGF-A, vascular endothelial growth factor A; MAPK8, mitogen-activated protein kinase 8; IL-6, interleukin 6; IL-12, interleukin 12; ERV, endogenous retrovirus; DNMTs, DNA methyltransferases; AP-1, activator protein 1; ISGs, interferon-stimulated genes; PARP, poly ADP-ribose polymerase; SASP, senescence-associated secretory phenotype; DCs, dendritic cells; ICAM1, intercellular adhesion molecule 1; ICD, immunogenic cell death; TNBC, triple-negative breast cancer; NFAT1, T cell nuclear factor 1; NSCLC, non-small-cell lung cancer; DNMT1, DNA methyltransferase 1; PROTACs, proteolysis-targeting chimeras; MDSCs, myeloid-derived suppressor cells; M-MDSCs, monocyte-type myeloid-derived suppressor cells; DAMPs, damage associated molecular patterns; MPNSTs, malignant peripheral nerve sheath tumors; PDAC, pancreatic ductal adenocarcinoma; MMPs, matrix metalloproteinases; SPOP, speckle-type POZ protein; IMC, immature myeloid cell; GCb, gemcitabine plus carboplatin; ICI, immune checkpoint inhibitors; irAEs, immune-related adverse events.
