# Supplementary material for: The Dual Effects of CDK4/6 Inhibitors on Tumor Immunity
Source: Cancers (Basel). 2025 Dec 15;17(24):3997. doi: 10.3390/cancers17243997 (PMC12730504; doi:10.3390/cancers17243997)
Supplement: Supplementary file 1 [file cancers-17-03997-s001.zip › cancers-4026110-supplementary.pdf]

Table S1. Summary of clinical trials on the combination of CDK4/6is and immunotherapy

NCT Number	Study Title	Study Status	Conditions	Interventions	Phases	Enrollment
NCT04887831	Trilaciclib, a CDK4/6 Inhibitor, in Patients with Advanced/Metastatic Bladder Cancer Receiving Chemotherapy Then Avelumab	Terminated	Urothelial Carcinoma, Bladder Cancer	Trilaciclib<br>Gemcitabine<br>Cisplatin/Carboplatin<br>Avelumab	Phase II	92
NCT05139082	Study of CDK4/6 Inhibitor Combined With PD-L1 Monoclonal Antibody in the Treatment of PD-1/PD-L1 Resistance and Abnormal Cell Cycle Digestive System Tumors	Unknown	Digestive System Tumors	TQB3616<br>TQB2450	Phase I/II	60
NCT05724355	CDK4/6 Inhibitor Plus Camrelizumab for PD-1 Inhibitor Refractory R/M NPC	Unknown	Nasopharyngeal Carcinoma	Dalpiciclib<br>Camrelizumab	Phase II	32
NCT03041311	Carboplatin, Etoposide, and Atezolizumab with or without Trilaciclib (G1T28), a CDK4/6 Inhibitor, in Extensive-Stage SCLC	Terminated	SCLC	Trilaciclib<br>Carboplatin<br>Etoposide<br>Atezolizumab	Phase II	107
NCT03601598	A Trial of SHR-1210 in Combination with SHR6390 in Patients with Advanced CRC, NSCLC and HCC	Unknown	CRC/HCC/NSCLC	SHR-1210<br>SHR6390	Phase I/II	41
NCT06364904	A Clinical Trail to Determine the Safety and Efficacy of the Combination of Tislelizumab with Cisplatin and Gemcitabine, with or without Trilaciclib for Patients with Untreated Unresectable and Metastatic Urothelial Carcinoma.	Not yet recruiting	Bladder Cancer	Tislelizumab<br>Cisplatin<br>Gemcitabine<br>Trilaciclib	Phase III	210
NCT06199271	Neoadjuvant Adebrelimab Plus Dalpiciclib in Head and Neck Squamous Cell Carcinoma	Not yet recruiting	Head and Neck Squamous Cell Carcinoma	Adebrelimab<br>Dalpiciclib	Phase II	30
NCT03294694	Ribociclib + PDR001 in Breast Cancer and Ovarian Cancer	Terminated	Metastatic HR+ /HER2− Breast Cancer Metastatic Ovarian Cancer	Ribociclib<br>PDR001<br>Fulvestrant	Phase I	33

NCT Number	Study Title	Study Status	Conditions	Interventions	Phases	Enrollment
NCT05935748	Ph2 Study NKT2152 with Palbociclib & Sasanlimab in Subjects with Advanced Clear Cell Renal Cell Carcinoma (ccRcc)	Active not recruiting	Advanced or metastatic ccRCC	NKT2152 (HIF1αi) Palbociclib Sasanlimab	Phase II	172
NCT04438824	Palbociclib and INCMGA00012 in People with Advanced Liposarcoma	Active not recruiting	Well-differentiated/Dedifferentiated Liposarcoma	INCMGA00012 Palbociclib	Phase II	42
NCT03498378	Avelumab, Cetuximab, and Palbociclib in Recurrent or Metastatic Head and Neck Squamous Cell Carcinoma	Active not recruiting	Head and Neck Squamous Cell Carcinoma	Avelumab Palbociclib Cetuximab	Phase I	24
NCT03573648	Neoadjuvant Endocrine Therapy, Palbociclib, Avelumab in Estrogen Receptor Positive Breast Cancer	Active not recruiting	Breast Cancer	Avelumab Endocrine therapy Palbociclib	Phase II	33
NCT06570031	ONO-4578-06: Phase I Study of ONO-4578 and Letrozole Plus CDK4/6 Inhibitors in Breast Cancer	Active not recruiting	HR+/HER2− Breast Cancer|	ONO-4578 Letrozole Palbociclib Abemaciclib	Phase I	46
NCT02778685	Pembrolizumab, Endocrine Therapy, and Palbociclib in Treating Postmenopausal Patients with Newly Diagnosed Metastatic Stage IV Estrogen Receptor Positive Breast Cancer	Active not recruiting	Metastatic Breast Carcinoma	Fulvestrant/Letrozole Palbociclib Pembrolizumab	Phase II	47
NCT05694871	Testing the Addition of Cemiplimab to Palbociclib for the Treatment of Advanced Dedifferentiated Liposarcoma	Active not recruiting	Advanced Dedifferentiated Liposarcoma	Palbociclib Cemiplimab	Phase II	77
NCT03147287	Palbociclib After CDK and Endocrine Therapy (PACE)	Active not recruiting	Metastatic Breast Cancer	Palbociclib Fulvestrant Avelumab	Phase II	220

NCT Number	Study Title	Study Status	Conditions	Interventions	Phases	Enrollment
NCT04075604	A Study of Neoadjuvant Nivolumab + Palbociclib + Anastrozole in Post-Menopausal Women and Men with Primary Breast Cancer	Completed	Breast Cancer	Nivolumab<br>Anastrozole<br>Palbociclib	Phase II	23
NCT03781960	Abemaciclib and Nivolumab for Subjects with Hepatocellular Carcinoma	Terminated	HCC	Abemaciclib<br>Nivolumab	Phase II	7
NCT04751929	Abemaciclib with or without Atezolizumab for mCRPC	Active not recruiting	Metastatic Castration-resistant Prostate Cancer	Abemaciclib<br>Atezolizumab	Phase II	75
NCT03997448	Abemaciclib and Pembrolizumab in Locally Advanced Unresectable or Metastatic Gastroesophageal Adenocarcinoma: Big Ten Cancer Research Consortium BTCRC-GI18-149	Terminated	Gastroesophageal Cancer	Pembrolizumab<br>Abemaciclib	Phase II	3
NCT02791334	A Study of Anti-PD-L1 Checkpoint Antibody (LY3300054) Alone and in Combination in Participants with Advanced Refractory Solid Tumors	Completed	Solid Tumor	LY3300054<br>Ramucirumab<br>Abemaciclib<br>Merestinib<br>LY3321367	Phase I	164
NCT03655444	Abemaciclib + Nivolumab in Patients with Recurrent/Metastatic Head and Neck Squamous Cell Carcinoma That Progressed or Recurred Within Six Months After Platinum-based Chemotherapy	Terminated	Head and Neck Squamous Cell Carcinoma	Abemaciclib<br>Nivolumab	Phase I/II	6
NCT02779751	A Study of Abemaciclib (LY2835219) in Participants with Non-Small Cell Lung Cancer or Breast Cancer	Active not recruiting	NSCLC<br>Breast Cancer	Abemaciclib<br>Pembrolizumab<br>Anastrozole	Phase I	100
NCT03938337	Abemaciclib and Pembrolizumab in Metastatic or Recurrent Head and Neck Cancer	Terminated	Head and Neck Cancer	Cohort 1: Not Previously Treated<br>Cohort 2: Treated Previously	Phase II	1

NCT Number	Study Title	Study Status	Conditions	Interventions	Phases	Enrollment
NCT03280563	A Study of Multiple Immunotherapy-Based Treatment Combinations in HR+/HER2− Breast Cancer	Completed	Breast cancer	Atezolizumab (MPDL3280A)<br>Bevacizumab<br>Exemestane<br>Fulvestrant<br>Ipatasertib<br>Tamoxifen<br>Abemaciclib	Phase I/II	138
NCT04213404	Ribociclib and Spartalizumab in R/M HNSCC	Unknown	HNSCC	Ribociclib<br>Spartalizumab	Phase I	13
NCT03484923	Study of Efficacy and Safety of Novel Spartalizumab Combinations in Patients with Previously Treated Unresectable or Metastatic Melanoma	Completed	Melanoma	PDR001<br>LAG525<br>INC280<br>ACZ885<br>LEE011	Phase II	196
NCT02978716	Trilaciclib (G1T28), a CDK4/6 Inhibitor, in Combination with Gemcitabine and Carboplatin in Metastatic Triple Negative Breast Cancer (mTNBC)	Terminated	mTNBC	Trilaciclib<br>Gemcitabine<br>Carboplatin	Phase II	102
NCT04000529	Phase Ib Study of TNO155 in Combination with Spartalizumab or Ribociclib in Selected Malignancies	Terminated	NSCLC<br>HNSCC<br>ESCC<br>Gastrointestinal Stromal Tumors<br>CRC	TNO155<br>Spartalizumab<br>Ribociclib	Phase I	122

Abbreviation: Hormone Receptor, HR; Human Epidermal Growth Factor Receptor 2, HER2; Small cell lung cancer, SCLC; Colorectal cancer, CRC; Hepatocellular carcinoma, HCC; Non-small cell lung cancer, NSCLC; Clear cell renal cell carcinoma, ccRCC; Hormone Receptor, HR; Castration-resistant prostate cancer, CRPC; Squamous cell carcinoma of the head and neck, HNSCC; Metastatic Triple-Negative Breast Cancer, mTNBC; Esophageal squamous cell carcinoma, ESCC;
